# Risk Factors Associated With Infection in Open Fractures of the Upper and Lower Extremities

**DOI:** 10.5435/JAAOSGlobal-D-20-00188

**Published:** 2020-12-08

**Authors:** Paul Tornetta, Gregory J. Della Rocca, Saam Morshed, Clifford Jones, Diane Heels-Ansdell, Sheila Sprague, Brad Petrisor, Kyle J. Jeray, Gina Del Fabbro, Sofia Bzovsky, Mohit Bhandari

**Affiliations:** From the Department of Orthopedic Surgery, Boston Medical Center, Boston, MA (Dr. Tornetta III); the University of Missouri School of Medicine, Columbia, MO (Dr. Della Rocca); the University of California San Francisco, San Francisco General Hospital, Orthopaedic Trauma Institute, San Francisco, CA (Dr. Morshed); the The CORE Institute, University of Arizona–Phoenix, Phoenix, AZ (Dr. Jones); the Department of Health Research Methods, Evidence, and Impact (Ms. Heels-Ansdell, Dr. Sprague, and Dr. Bhandari) and the Division of Orthopaedic Surgery, Department of Surgery (Dr. Sprague, Dr. Petrisor, Ms. Del Fabbro, Ms. Bzovsky, and Dr. Bhandari), McMaster University, Hamilton, Ontario, Canada; and the Department of Orthopaedic Surgery, Prisma Health-Upstate, Greenville, SC (Dr. Jeray).

## Abstract

**Introduction:**

Open fractures are associated with a high risk of infection. The prevention of infection is the single most important goal, influencing perioperative care of patients with open fractures. Using data from 2,500 participants with open fracture wounds enrolled in the Fluid Lavage of Open Wounds trial, we conducted a multivariable analysis to determine the factors that are associated with infections 12 months postfracture.

**Methods:**

Eighteen predictor variables were identified for infection a priori from baseline data, fracture characteristics, and surgical data from the Fluid Lavage of Open Wounds trial. Twelve predictor variables were identified for deep infection, which included both surgically and nonoperatively managed infections. We used multivariable Cox proportional hazards regression analyses to identify the factors associated with infection. Irrigation solution and pressure were included as variables in the analysis. The results were reported as adjusted hazard ratios (HRs), 95% confidence intervals (CIs), and associated *P* values. All tests were two tailed with alpha = 0.05.

**Results:**

Factors associated with any infection were fracture location (tibia: HR 5.13 versus upper extremity, 95% CI 3.28 to 8.02; other lower extremity: HR 3.63 versus upper extremity, 95% CI 2.38 to 5.55; overall *P* < 0.001), low energy injury (HR 1.64, 95% CI 1.08 to 2.46; *P* = 0.019), degree of wound contamination (severe: HR 2.12 versus mild, 95% CI 1.35 to 3.32; moderate: HR 1.08 versus mild, 95% CI 0.78 to 1.49; overall *P* = 0.004), and need for flap coverage (HR 1.82, 95% CI 1.11 to 2.99; *P* = 0.017).

**Discussion:**

The results of this study provide a better understanding of which factors are associated with a greater risk of infection in open fractures. In addition, it can allow for surgeons to better counsel patients regarding prognosis, helping patients to understand their individual risk of infection.

Open fractures are associated with a high risk of infection.^1,2,3,4,5,6,7,8^ Infection of an open fracture in the upper or lower extremities is associated with poorer outcomes, including increased rates of delayed union and nonunion, increased length of hospital stay, and lower health-related quality of life.^1,3,8^ The results from the Fluid Lavage of Open Wounds (FLOW) trial have confirmed that patients who had an infection or another complication that required an additional surgery reported notably lower physical and mental health-related quality of life in the 12 months after their fracture compared with those who did not have an infection.^9^ The sequelae of infections continue to be a cause of prolonged morbidity, prolonged hospitalization, and even death.^10^

Although previous investigations being conducted on the predictive risk factors for infection, none have evaluated a large multicenter data set.^11,12,13,14,15^ The purpose of this study was to determine the factors associated with infection in a large prospective series of open fractures of the upper and lower extremities treated at multiple centers.

## Methods

### Fluid Lavage of Open Wounds Trial

The FLOW trial was a multicenter, blinded, randomized controlled trial, using a 2 × 3 factorial design that evaluated irrigation solution (soap versus normal saline) and irrigation pressure (very low versus low versus high) in participants with open fracture wounds.^9^ Research ethics board approval for the FLOW trial was obtained at the coordinating center (McMaster University) (REB: 08-268) and at each participating site. The trial was registered at clinicaltrials.gov (Clinical trials identification number: NCT00788398).

Participants were randomized using an internet-based randomization system, which ensured concealed randomization of eligible consenting patients. Participants were followed for 12 months from their injury for assessment of clinical outcomes. The primary outcome in FLOW was a composite of reoperation, defined as surgery that occurred within 12 months after the initial procedure to treat an infection at the surgical site or contiguous to it, manage a wound-healing problem, or promote bone healing. Secondary clinical outcomes included nonoperatively managed infections, wound, or fracture healing complications. All clinical outcomes were adjudicated by an independent Adjudication Committee.

The trial included 2,447 participants across 41 clinical sites in the United States, Canada, Australia, Norway, and India. The FLOW primary analysis found soap to have a notably higher reoperation rate than saline and found no differences between the three irrigation pressures evaluated.^16^

### Selection of Predictor Variables

Based on biologic rationale and previous reports in the literature, we identified potential predictor variables a priori from the baseline, fracture characteristics, and surgical data collected as part of the FLOW trial. These were chosen by the FLOW Steering Committee based on previous literature and experience. We identified 18 predictor variables for infection within 12 months (Table 1), and 12 predictor variables for deep infection, including both surgically and nonoperatively managed infections (Table 2). It was methodologically necessary to include both irrigation pressure and solution as variables in all analyses because they were the basis of the initial FLOW trial. When selecting our predictor variables for the analysis, we ensured that at least 10 events were found for each parameter to avoid having an over fitted or unstable mode.^17^

**Table 1:** Factors Associated With Infection in Open Fractures (n = 2,338; 289 events)

Independent Variable	Incidence of Predictors, n (%)	Adjusted HR (95% CI)	*P* Value
Fracture location			<0.001
Tibia	883 (37.8)	5.13 (3.28-8.02)	
Other lower extremity	726 (31.1)	3.63 (2.38-5.55)	
Upper extremity	729 (31.2)	1.00	
Low-energy mechanism of injury	280 (12.0)	1.63 (1.08-2.46)	0.019
Degree of contamination			0.004
Mild	1,799 (76.9)	1.00	
Moderate	416 (17.8)	1.08 (0.78-1.49)	
Severe	123 (5.3)	2.12 (1.35-3.32)	
Age (10-yr increase)	45.1 (17.8) mean (SD)	1.04 (0.96-1.12)	0.376
Male sex	1,622 (69.4)	1.05 (0.79-1.39)	0.733
Current smoker	754 (32.2)	1.08 (0.84-1.40)	0.537
Other major injury^a^	722 (30.9)	0.91 (0.69-1.19)	0.496
Comminuted or segmental fracture	1,579 (67.5)	1.21 (0.91-1.60)	0.182
Bone loss	512 (21.9)	1.19 (0.90-1.58)	0.223
Method of definitive fixation			0.148
Nail	792 (33.9)	1.00	
Plate	1,177 (50.3)	1.36 (0.997-1.86)	
Other	369 (15.8)	1.30 (0.86-1.98)	
Bone grafting at initial surgery	50 (2.1)	0.95 (0.35-2.60)	0.918
Type III postoperative Gustilo type	846 (36.2)	1.23 (0.92-1.64)	0.161
Total operating time ≥120 min	997 (42.6)	1.11 (0.86-1.44)	0.429
Time to first incision from injury			0.126
<6 hr	465 (19.9)	1.00	
6-12 hr	980 (41.9)	0.92 (0.68-1.23)	
>12-24 hr	785 (33.6)	0.71 (0.50-1.02)	
>24 hr	108 (4.6)	1.27 (0.66-2.43)	
Delayed wound closure (wound not closed at initial irrigation and débridement)	373 (16.0)	0.95 (0.66-1.38)	0.796
Randomized solution			0.922
Soap	1,178 (50.4)	1.1 (0.80-1.28)	
Saline	1,160 (49.6)	1.00	
Randomized pressure			0.833
High	784 (33.5)	1.00	
Low	772 (33.0)	1.05 (0.79-1.41)	
Very low	782 (33.4)	1.09 (0.82-1.46)	
Time-dependent variables			
Wound flap	108 (4.6)	1.82 (1.11-2.99)	0.017

CI = confidence interval, HR = hazard ratio

a At least one of the following: femoral fracture, pelvic fracture, spinal fracture, liver injury, bowel injury, splenic injury, other abdominal injury, hemo/pneumothorax, closed head injury, urogenital injury, traumatic amputation, vascular injury, lung contusion, thoracic injury, hip fracture, and spinal injury.

**Table 2:** Factors Associated With Deep Infection in Open Fractures (n = 2,346; 156 events)

Independent Variable	Incidence of Predictors, n (%)	Adjusted HR (95% CI)	*P* Value
Fracture location			<0.001
Tibia	885 (37.7)	2.72 (1.57-4.71)	
Other lower extremity	729 (31.1)	2.98 (1.72-5.18)	
Upper extremity	732 (31.2)	1.00	
Delayed wound closure (wound not closed at initial irrigation and débridement)	380 (16.2)	1.89 (1.24-2.90)	0.003
Type III postoperative Gustilo type	852 (36.3)	1.57 (1.09-2.27)	0.016
Age (10-yr increase)	45.1 (17.7) mean (SD)	1.07 (0.96-1.18)	0.220
Male sex	1,626 (69.3)	0.92 (0.64-1.33)	0.663
Current smoker	758 (32.3)	1.03 (0.73-1.47)	0.855
Other major injury^a^	724 (30.9)	1.03 (0.72-1.45)	0.892
Total operating time ≥120 min	1,000 (42.6)	0.98 (0.69-1.39)	0.921
Time to first incision from injury			
<6 hr	467 (19.9)	1.00	
6-12 hr	985 (42.0)	0.77 (0.52-1.13)	0.083
>12-24 hr	786 (33.5)	0.54 (0.34-0.87)	
>24 hr	108 (4.6)	0.88 (0.36-2.16)	
Randomized solution			
Soap	1,181 (50.3)	0.99 (0.72-1.36)	0.955
Saline	1,165 (49.7)	1.00	
Randomized pressure			
High	787 (33.5)	1.00	
Low	774 (33.0)	1.10 (0.75-1.62)	0.817
Very low	785 (33.5)	0.98 (0.66-1.46)	
Time-dependent variables			
Wound flap	110 (4.7) ever	2.05 (1.14-3.71)	0.017

CI = confidence interval, HR = hazard ratio

a At least one of the following: femoral fracture, pelvic fracture, spinal fracture, liver injury, bowel injury, splenic injury, other abdominal injury, hemo/pneumothorax, closed head injury, urogenital injury, traumatic amputation, vascular injury, lung contusion, thoracic injury, hip fracture, and spinal injury.

### Definition of Infection

Infection in participants was defined as a constellation of clinical symptoms and laboratory examinations and classified according to the Center for Disease Control criteria.^10^ These included, but were not limited to, fever, erythema/cellulites, positive tissue cultures, and frank purulent drainage occurring within 12 months from the initial procedure. When interpreting the criteria, any infections that were superficial to the fascia were considered to be “superficial incisional surgical site infections,” and any infections that were deep to the fascia were considered to be “deep incisional surgical site infection” (including infections of the bone [osteomyelitis]). The central adjudication committee adjudicated all reported infections to determine whether they met the Center for Disease Control criteria and classified them as superficial or deep.

### Data Analysis—Predictors of Infection and Deep Infection

We conducted two multivariable Cox proportional hazards regression analyses with time to any infection and time to deep infection as the dependent variables to identify the factors associated with the outcome. Participants with complete data for all selected predictor variables were included in the analyses. The results were reported as adjusted hazard ratios (HRs), 95% confidence intervals (CIs), and associated *P* values. All tests were two tailed with alpha = 0.05.

## Results

### Participant Characteristics

Seven participants with delayed definitive fixation who experienced an infection before definitive fixation were excluded from the analysis. This left a total of 2,338 participants with complete data for the selected predictor variables who were included in the Cox proportional hazards regression analysis with time to infection as the independent variable. Their mean age was 45.1 years. Most were men (69.4%) and had a lower extremity fracture (68.8%) (Table 1). Of the participants included in this analysis, 289 (12.4%) experienced either a superficial or deep infection within 12 months of initial surgery and 156 (6.7%) experienced a deep infection during that time.

### Predictors of Any Infection

The factors associated with any infection were fracture location (tibia: HR 5.13 versus upper extremity, 95% CI 3.28 to 8.02; other lower extremity: HR 3.63 versus upper extremity, 95% CI 2.38 to 5.55; overall *P* < 0.001), low energy injury (HR 1.63, 95% CI 1.08 to 2.46; *P* = 0.019), degree of wound contamination (severe: HR 2.12 versus mild, 95% CI 1.35 to 3.32; moderate: HR 1.08 versus mild, 95% CI 0.78 to 1.49; overall *P* = 0.004), and need for flap coverage (HR 1.82, 95% CI 1.11 to 2.99; *P* = 0.017) (Table 1).

### Predictors of Deep Infection

The factors associated with deep infection were location (tibia: HR 2.72 versus upper extremity, 95% CI 1.57 to 4.71; other lower extremity: HR 2.98 versus upper extremity, 95% CI 1.72 to 5.18; overall *P* < 0.001), Gustilo Type III fracture (HR 1.57, 95% CI 1.09 to 2.27; *P* = 0.016), delayed wound closure (wound not closed at initial irrigation and débridement) (HR 1.89, 95% CI 1.24 to 2.90; *P* = 0.003), and need for flap coverage (HR 2.05, 95% CI 1.14 to 3.71; *P* = 0.017) (Table 2).

## Discussion

Our analysis of FLOW data found that patients with lower extremity fractures, especially tibia fractures, compared with upper extremity fractures, were at a higher risk of infection. In addition, high energy injuries, wounds with severe and moderate contaminations, and wounds that required flap coverage were associated with infection. Factors associated with deep infections included lower extremity factors, especially tibia fractures, compared with upper extremity fractures, Gustilo Type III injuries, fractures requiring delayed wound closure (wound not closed at initial irrigation and débridement), and the need for flap coverage. Interestingly, we did not find any patient factors (age, sex, and smokers) or treatment factors (method of fixation and timing of surgery) to be predictive of infection. All predictive factors were related to the severity of the injury.

Participants with a lower extremity open fracture compared with those with an upper extremity open fracture were found to be at an increased risk of any infection and of a deep infection. These findings coincide with a recent meta-analysis that examined fracture location in two randomized controlled trials, seven prospective, and 18 retrospective studies.^8^ The study found that lower extremity fractures were at a notably higher risk of developing infectious complications (11.8% versus 5.4%; risk ratio (RR) 1.94, *P* < 0.0001).^8^ Moreover, a retrospective study found that the tibia had an increased odds ratio for infection of 2.44 (95% CI 1.26 to 4.73) when compared with nontibial injury.^12^

As expected, we found that worse injuries have higher complication rates. As demonstrated in other investigations, we were unable to find an association between infection and patient factors (age, sex, and smokers) nor treatment factors (method of fixation and timing of surgery).^12,18-20^ In a prospective study of 480 participants, the investigators did not find smoking status to be a statistically notable factor for infection.^4^ Furthermore, a retrospective study of 1,043 participants with an open fracture of the ankle did not find a statistically notable association between smoking and postoperative infection.^21^ Similarly, a retrospective review of 478 patients with open ankle fractures showed no correlation between patient-reported smoking and wound complications, including infection, after ankle fracture surgery.^22^

Fractures managed with flaps were at a higher risk of infection that is similar to other studies.^23-25^ Furthermore, delayed wound closure was found to be associated with deep infection. These findings are likely driven by the severity of the injury because simpler wounds are closed initially.

To our knowledge, few studies have prospectively enrolled large numbers of open fractures, particularly including upper extremity injuries. The primary strength of this study is the preplanned analysis of 2,338 open fractures from 41 clinical sites in the United States, Canada, Australia, Norway, and India, giving this secondary analysis a more robust basis for predicting infection and secondary intervention. The large sample size and diversity of participants allows for greater generalizability of our findings and increases the external validity of our results. Furthermore, the use of the Cox proportional hazards regression allowed for the control of potentially confounding variables.

However, this study has several limitations. First, because of missing data, it was not feasible to include all 2,447 participants from the FLOW trial in this analysis. In addition, this analysis was limited by the variables collected as part of the FLOW trial. As a result, some factors potentially associated with infection may not have been collected and were not included in this analysis.

In conclusion, recognizing risk factors associated with infections in open fractures is important in preventing these infections and other complications that impede the healing process. This data set gives us more information to risk adjust and understand the factors leading to infection in open fractures. These findings can allow surgeons to better advise patients about treatment and prognosis and set appropriate expectations. Future research should focus on exploring methods to reduce infection in this high risk cohort. Finally, the results of this study may inform future research by identifying a need for large, prospective research, particularly on the lower extremities.

## Appendix 1 FLOW Investigators

**Steering Committee:** Mohit Bhandari (Chair, McMaster University), Gordon H. Guyatt (Co-Chair, McMaster University), Kyle J. Jeray (Co-Chair, Greenville Health System), Stephen D. Walter (McMaster University), Brad Petrisor (McMaster University), Emil H. Schemitsch (St. Michael's Hospital), Paul Tornetta III (Boston University Medical Center), Jeff Anglen (Eskenazi Health Services, Indiana University), Michael Bosse (Carolinas Health Care System), Susan Liew (The Alfred), Parag Sancheti (Sancheti Institute for Orthopaedics and Rehabilitation)

**Global Methods Center:** Mohit Bhandari (Principal Investigator); Sheila Sprague (Research Methodologist); Paula McKay, Kim Madden, Kerry Tai (Project Management); Diane Heels-Ansdell, (Statistical Analysis); Lisa Buckingham, Aravin Duraikannan (Data Management) (McMaster University)

**United States Methods Center:** Kyle J. Jeray (Principal Investigator), Stephanie L. Tanner, Rebecca G. Snider (Project Management) (Greenville Health System)

**Data Monitoring Committee:** Douglas Altman (University of Oxford), Rajiv Gandhi (Toronto Western Hospital), Markus Bischoff (McMaster University)

**Adjudication Committee:** Mohit Bhandari (McMaster University), Gregory J. Della Rocca (University of Missouri Health Care), Brad Petrisor (McMaster University), Kyle J. Jeray (Greenville Health System), Emil H. Schemitsch (St. Michael's Hospital)

**Participating Clinical Sites:**

Canada—Hamilton Health Sciences—Brad Petrisor, Bill Ristevski, Krishan Rajaratnam, Dale Williams, Matthew Denkers, Drew Bednar, John Sadler, Desmond Kwok, Mohit Bhandari, Brian Drew, Ivan Wong, Paula McKay, Kim Madden, Kerry Tai. St. Michael's Hospital—Jeremy A. Hall, Michael D. McKee, Emil H. Schemitsch, Henry Ahn, Daniel Whelan, James Waddell, Timothy Daniels, Earl Bogoch, Aaron Nauth, Milena R. Vicente, Jennifer T. Hidy. London Health Sciences Centre—David Sanders, Abdel-Rahman Lawendy, Kevin Gurr, Timothy Carey, Chris Bailey, Mark Macleod, Debra Bartley, Christina Tieszer. Queen Elizabeth II Health Sciences Centre—Chad Coles, Ross Leighton, C. Glen Richardson, Michael Biddulph, Michael Gross, Michael Dunbar, J. David Amirault, David Alexander, Catherine Coady, Mark Glazebrook, David Johnston, William Oxner, J. Andrew Trenholm, Gerald Reardon, Kelly Trask, Shelley MacDonald. The Ottawa Hospital Civic Campus—Steven Papp, Wade Gofton, Allan Liew, Stephen Kingwell, Joseph O'Neill, Garth Johnson, Eugene Wai, Julia Foxall. Vancouver General Hospital—Henry M. Broekhuyse, Peter J. O'Brien, Piotr A. Blachut, Kelly A. Lefaivre, Raman Johal. Hôpital du Sacré-Coeur de Montréal—Stéphane Leduc, G. Yves Laflamme, Pierre Beaumont, Michel Malo, Benoit Benoit, Dominique Rouleau, Pierre Ranger, Julie Fournier, Karine Tardif. McGill University Health Centre—Rudy Reindl, Greg Berry, Edward Harvey, William Fisher, Mark Burman, Paul Martineau, Eric Lenczner, Robert Marien, Robert Turcotte, Michael Tanzer, Max Talbot, Peter Jarzem, Mike Weber, Fiona Houghton. University of British Columbia/Fraser Health Authority—Robert McCormack, Kelly Apostle, Dory Boyer, Farhad Moola, Bertrand Perey, Trevor Stone, Darius Viskontas, H. Michael Lemke, Mauri Zomar, Karyn Moon, Raely Moon. Sunnybrook Health Sciences Centre—Hans Kreder, Richard Jenkinson, David Stephen, Markku Nousiainen, Terry Axelrod, Veronica Wadey, Michael Ford, Joel Finkelstein, Richard Holtby, Robin Richards, Sebastian Rodriguez-Elizalde, Diane Nam, Albert Yee, Patrick Henry, John Murnaghan, Harsha Malempati, Julian Sernik, Tim Dwyer, Katrine Milner, Monica Kunz, Melanie MacNevin, Wesley Ghent, Fathima Adamsahib, Ria De Gorter, Michelle Arakgi. Winnipeg Health Sciences Centre—Ted V. Tufescu, Brad Pilkey, Chris Graham, Laurie Barron, Allan Hammond, Nigar Sultana. Queen's University—Ryan T. Bicknell, David Pichora, Aaron Campbell, Fiona Howells. Centre de Recherche du Centre Hospitalier Universitaire de Sherbrooke (CRCHUS)—Annie Deshaies, Frédéric Balg, François Cabana, Rejean Dumais, Jean-François Joncas, Marc-André Magalhaes-Grave, Nicolas Patenaude, Bernard LaRue, Stéphane Ricard, Chantal Théorêt, François Vézina, Amy Svotelis, Jennifer Downey. Hôpital de l'Enfant-Jésus—Stéphane Pelet, Jean Lamontagne, Luc Bédard, Alexandre Denault, Pierre Lavallée, Luc Petitclerc, Bernard Laliberté, Martin Bédard, Marie-Eve Roger, Luc Lemire, Hélène Côté, Linda Lépine, Pascale Lévesque-Bernier.

United States—Greenville Health System—Kyle J. Jeray, J. Scott Broderick, David R. Goetz, Thomas M. Schaller, Scott E. Porter, Michael L. Beckish, John D. Adams, Jr, Benjamin B. Barden, Grant W. Bennett, David M. Conner, Aaron T. Creek, Melissa M. Earles, Stephen H. Finley, Jonathan L. Foret, Garland K. Gudger, Jr, Richard W. Gurich, Jr, Austin D. Hill, S. Matthew Hollenbeck, Lyle T. Jackson, Benjamin S. Koch, Kevin K. Kruse, Wesley G. Lackey, Justin W. Langan, Julia Lee, Lauren C. Leffler, Michael J. Maughon, Jr, S. Brennan McClure, Timothy J. Miller, R. Lee Murphy, Jr, Lawrence K. O'Malley, Dustin M. Price, Lorra M. Sharp, J. Adam Smitherman, John A. Tanksley, Jr, Erick G. Torres, Dylan J. Watson, Scott T. Watson, Stephanie L. Tanner, Rebecca G. Snider, Shea A. Bielby, Lauren A. Nastoff, Robert J. Teasdall. United States Army Institute of Surgical Research and Brooke Army Medical Center—Joseph Hsu, Katherine M. Bedigrew, Tod Gerlinger, Dave Brown, Joseph Alderete, Kevin Kirk, Mickey Cho, Anthony Johnson, Raymond Topp, Damian Rispoli, James Ficke, Eric Ritchie, Anthony Beardmore, Siraj Sayeed, Michael Charlton, Kristen Walick, Dmitry Tuder, Greg Maytok, Travis Burns, Donald Gajewski, Warren Kactmas, Ramnov Andreson, Patrick Osborn, Michael Connally, Donna Lopez, Mary Fan, Dennis Mann, Andrea Garza, Rina L. Harman. Duke University Medical Center—Steven Olson, Robert Zura, Rachel Reilly, Prerana Patel, Claude T Moorman, Fraser Leversedge, Chard Harbour, Brian Brigman, David Ruch, Nikoletta Leontaritis, Michael Bolognesi, Shalini Ramasunder, Alison Toth, Allen Diane, Grant Garrigues, Dean Taylor, Richard C. Mather III, Kristoff Reid, Robert Lark, Samuel Adams, Maria Manson. San Francisco General Hospital—Utku Kandemir, Saam Morshed, Murat Pekmezci, Richard Coughlin, Trigg McClellan, Meir Marmor, Eric Meinberg, Tigist Belaye, Jonathan Kwong. Orthopaedic Associates of Michigan—Clifford B. Jones, James R. Ringler, Terrence J. Endres, David J. Bielema, Michael R. Jabara, Samuel G. Agnew, Debra L. Sietsema, Jane E. Walker. University of Missouri Health Care—Gregory J. Della Rocca, Brett D. Crist, Yvonne M. Murtha, David A. Volgas, James P. Stannard, Linda K. Anderson, Kelly M. Sullivan, Lori Kramer Clark, Kathleen Markley, Stacee Clawson. Hennepin County Medical Center—Andrew Schmidt, Patrick Yoon, Thomas Varecka, Matthew Karam, Jerald R. Westberg. St. Louis University—Lisa K. Cannada, Jason Stoneback, Kevin Kuhn, Erik Nott, Leslie Dillender. Eskenazi Health Services, Indiana University—Karl Shively, Brian Mullis, Janos Ertl, Ripley Worman, Jeffrey Anglen, Valda Frizzell, Molly Moore. Miami Valley Hospital—Michael J. Prayson, David Nelles, Jason Vourazeris, Matthew Ross, Richard T. Laughlin, Joseph Cox, Roman Trimba, Joy M. Bradford-Johnson. Lahey Clinic—Andrew J. Marcantonio, Michael Kain, Richard Wilk, Mark Lemos, Joshua Baumfeld, John Tilzey, Brian Jolley, John Garfi. University of Pittsburgh Medical Center—Ivan Tarkin, Andrew Evans, Peter Siska, Lisa Blackrick, Dana J. Farrell. University of Alabama at Birmingham—Emily Keener, Jason Lowe, William Min, Jeffrey Leary, Rena Stewart, David Volgas, Leslie Barnes, Nurit Shadmi, Matthew Robinson, Taylor Vlack, Kathryn Hornbuckle, Melanese Leonard, Nikia Hawkins Malone, Tanya Nix, Jessica Goldstein. University of California Irvine—David Zamorano, Martin Tynan, Samuel Bederman, Nitin Bhatia, Arthur Kreitenberg, Bang Hoang, Deeba Pourmand, Deanna Lawson. Scottsdale Healthcare—Anthony Rhorer, Brian Miller, Gilbert Ortega, Lori Wood, Veronica Place.

International—The Alfred (Australia)—Susan Liew, Harvinder Bedi, Ashley Carr, Andrew Chia, Hamish Curry, Steve Csongvay, Craig Donohue, Stephen Doig, Elton Edwards, Eugene Ek, Max Esser, Greg Etherington, Richard Freeman, Andrew Gong, Doug Li, Matthan Mammen, Russell Miller, Ash Moaveni, Mathias Russ, Lu Ton, Tom Treseder, Otis Wang, Zoe Murdoch, Claire Sage, Adam Dowrick. Oslo University Hospital (Norway)—John Clarke-Jenssen, Frede Frihagen, Lars Nordsletten, Tor Nicolaysen, Hilde Apold, Petter Iversen, Are Stodle, Mette Andersen, Vera Halvorsen, Geir Hjorthaug, Anders Lippert, Ida Sletten, Ellen Langslet, Marius Molund, Asgeir Amundsen, Oliver Muller, Cathrine Aga, Torben Ianssen, Gunnar Flugsrud, Jonas Rydinge, Kim Hemlock, Jan Egil Brattgjerd, John Magne Hoseth, Bernhard Flatoy, Havard Furunes, Peder Bogsti, Guri Ekås, Gilbert Moatshe, Ali Al-Ashtari, Tore Fjalestad, Fredrik Nilsen, Morten Smedsrud, Anne Christine Brekke, Elise Berg Vesterhus, Sissel Knuts. Sancheti Institute for Orthopaedics and Rehabilitation (India)—Parag Sancheti, Steve Rocha, Chetan Puram, Atul Patil, Neelam Jhangiani. Highway Hospital (India)—Anil K. Rai, Kamal Narayan Rai. Jabade Hospital (India)—Vivek V. Jabade, Deepali Nassikars. Karne Hospital (India)—Narayan J Karne, Chetan Metha. Apollo Hospital Tondierpet (India)—A Navaladi Shankar. Apollo Hospital Greams Lane (India)—A Navaladi Shankar, R Saravana. Nirmal Hospital (India)—Ajay Gupta, Neeraj Jain. RLB Hospital and Research Centre (India)—Mahesh Bhatia, Vinod Arora, Vivek Tyagi, Anoop Dubey. Popular Hospital (India)—Vinit Yadav, Rani Rai. Kolhapur Institute of Orthopaedics and Trauma (India)—Kiran M. Doshi, Arjun Patil.
